# Type II halogen···halogen contacts are halogen bonds

**DOI:** 10.1107/S205225251303491X

**Published:** 2013-12-31

**Authors:** Pierangelo Metrangolo, Giuseppe Resnati

**Affiliations:** a Laboratory of Nanostructured Fluorinated Materials of the Department of Chemistry, Materials and Chemical Engineering "Giulio Natta", Politecnico di Milano, Via L. Mancinelli 7, Milano, 20131, Italy

**Keywords:** halogen bonds, halogenated phenols

## Abstract

Cl/Br/I alternative substitutions in a series of dihalophenols indicate that type I and type II halogen···halogen contacts have different chemical nature. Only the latter ones qualify as true halogen bonds, according to the recent IUPAC definition.

Halogenated phenols (HPs) are naturally present in the marine environment (Gribble, 2010 ▶). They are also important man-made compounds owing to their chemical inertness and applications in many industrial processes. For instance, chlorinated phenols, *e.g.* 2,4,6-trichlorophenol and its isomers, have been routinely used as pesticides, bactericides and fungicides, and a number of tribromophenols are commercially produced as flame retardants and wood preservatives.

The carbon–halogen bond can be quite stable in the environment and HPs are recalcitrant molecules, widespread organic pollutants found in air, water, soil and sediments (Shao *et al.*, 2011 ▶). The HPs mentioned above have, in fact, been detected and quantified in blood from birds, fish and mammals, including humans. The retention of HPs by living organisms is related to their structural resemblance to the thyroid hormones 3,3′,5,5′-tetraiodo-l-thyroxin (thyroxine, T4) and 3,3′,5-triiodo-l-thyronine (T3). In fact, *para*- and *meta*-halophenols bind to some proteins such as transthyretin (TTR), thyroxin-binding globulin (TGB) and albumin (ALB), and the receptor affinity of *o,o’*-dihalophenols is remarkably high (Kitamura *et al.*, 2008 ▶).

To understand the recognition features of HPs at molecular level is thus of paramount importance as it may help to rationalize the adverse biological effects of HPs to which humans are commonly exposed. Many drugs are halogenated and ascertaining the generalities of the active role of halogen atoms in determining specific interactions may help to make drug design more rational and effective.

HPs are very interesting tectons in crystal engineering and this adds extra relevance to the study of the landscape of interactions they form. HPs possess manifold binding sites for molecular recognition. For example, a monohalo-phenol may potentially receive electron density at the phenolic hydrogen (*i.e.* hydrogen bonding) and along the extension of the C–halogen bond (*i.e.* halogen bonding). Moreover, the phenolic oxygen lone pairs may accept hydrogen and halogen bonds, as well as the halogen atom can do in a direction perpendicular to the C–halogen bond. Finally, halogen atoms can form halogen···halogen contacts, which are routinely divided into two major categories depending on their geometry, *i.e.* type I (*θ*_1_ ≃ *θ*_2_) and type II (*θ*_1_ ≃ 180°, *θ*_2_ ≃ 90°), where *θ*_1_ and *θ*_2_ are the C—*X*···*X*′ and C—*X*′···*X* angles (*X*, *X*′ = Cl, Br, I) (Desiraju & Parthasarathy, 1989 ▶, see also Fig. 1 ▶).

The geometric difference between type I and II halogen···halogen contacts is due to their chemical difference. In fact, type II contacts involve an approach of the electrophilic region of one halogen atom to the nucleophilic region of the other, while in type I contacts two halogen atoms minimize repulsion by interfacing the neutral regions of their electrostatic potential surfaces. In their paper in this issue of the journal, Mukherjee & Desiraju (2014 ▶) unambiguously demonstrate that this is indeed the case. By exploiting the greater size and polarizability of I and Br *versus* Cl in a series of dihalophenols, they prove the fundamental difference between type I and II *X*···*X* contacts.

This study is timely in light of the recent IUPAC definition of the halogen bond that states that: ‘A halogen bond occurs when there is evidence of a net attractive interaction between an electrophilic region associated with a halogen atom in a molecular entity and a nucleophilic region in another, or the same, molecular entity’ (Desiraju *et al.*, 2013 ▶). Based on the observations reported by Mukherjee & Desiraju, among others, only type II halogen···halogen contacts qualify as being true halogen bonds.

In order to demonstrate this, Mukherjee & Desiraju studied a variety of simple dihalophenols. In the crystal structure of 3,4-dichlorophenol (**1**), type I and type II Cl···Cl contacts coexist with the O—H···O hydrogen bond, which is the strongest interaction possible in this structure. This is a very rare phenomenon as strong interactions (like O—H···O) often suppress the subtle differences in the weak interactions. This rare feature makes 3,4-dichlorophenol an ideal candidate for a Cl/Br/I alternative substitution strategy.

4-Bromo-3-chlorophenol (**2**) is isostructural to 3,4-dichlorophenol with the Br involved in a type II contact (*i.e.* halogen bond), while the Cl is involved in a type I contact. On the other hand, 3-bromo-4-chlorophenol (**3**) shows a quite different packing where the Br is halogen-bonded to the O atom and the Cl is involved in a type I contact. Just a single atom mutation (*i.e.* 3-Cl → 3-Br) completely upsets the structure of 3,4-dichlorophenol. 3-Iodo-4-chlorophenol (**4**) and 3,5-dibromophenol (**5**) are structurally very similar to 3-bromo-4-chlorophenol, highlighting the higher tendency of the heavier halogens towards halogen bonds. The electrophilic nature of Br and its propensity for the formation of type II contacts has also been corroborated by crystal prediction analysis, which highlighted the significant differences in the overall features of the structural landscapes of 4-bromo-3-chlorophenol and 3-bromo-4-chlorophenol. Cl···Cl and Br···Br contacts in (**1**) and (**2**) were also analyzed by variable-temperature (VT) single-crystal X-ray crystallography. The VT study revealed that, in both (**1**) and (**2**), the percentage increase in the *X*···*X* distances upon raising the temperature is greater for type II than type I contacts. This is consistent with the electrostatic nature (*i.e.* effective at shorter range) of type II interactions compared with the weak van der Waals nature of type I.

Importantly, Mukherjee & Desiraju demonstrate that the unusual elastic bending shown by crystals of 4-bromo-3-chlorophenol (**2**) upon application of mechanical stress may be related to the type II Br···Br interactions in (**2**). These interactions are stronger than the corresponding type II Cl···Cl ones in (**1**) and this energy difference leads to a standard mechanical behavior, *i.e.* plastic deformation. The observed correlation between the strengths of the respective halogen bonds and the solid-state properties of the studied materials is extremely important. Functional materials design using halogen bonds is still in its infancy, and this study may be taken as a good starting model for material property design. The paper by Mukherjee & Desiraju gives a major contribution to the understanding of the relative relevance of the different interactions potentially formed by halogen atoms. If their chemical nature, strength and directionality are mastered, major advantages may clearly result.

## Figures and Tables

**Figure 1:**
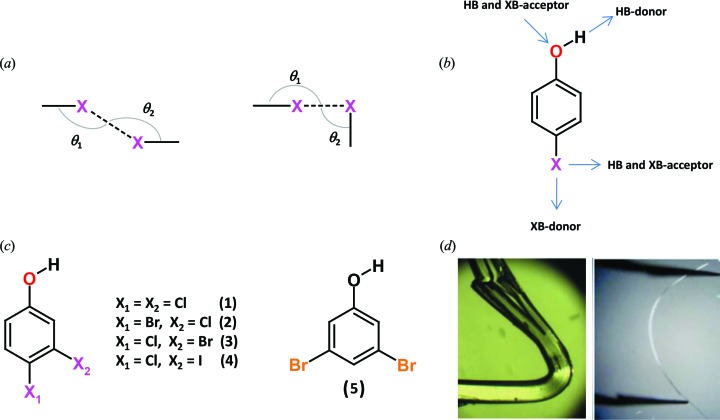
(*a*) Different approach geometries in type I (left) and type II (right) halogen···halogen contacts; (*b*) different binding sites of a 4-halophenol for hydrogen and halogen-bond-based intermolecular recognition; (*c*) chemical structures of the compounds studied by Mukherjee & Desiraju; (*d*) plastic and elastic bending shown by (**1**) (left) and (**2**) (right), respectively.
